# Exogenous nitric oxide stimulates early egress of *Eimeria tenella* sporozoites from primary chicken kidney cells *in vitro*

**DOI:** 10.1051/parasite/2021007

**Published:** 2021-02-12

**Authors:** Xinlei Yan, Wenying Han, Xianyong Liu, Xun Suo

**Affiliations:** 1 Food Science and Engineering College of Inner Mongolia Agricultural University Hohhot 010018 China; 2 State Key Laboratory of Agrobiotechnology, National Animal Protozoa Laboratory, College of Veterinary Medicine, China Agricultural University Beijing 100193 China

**Keywords:** Egress, *Eimeria tenella* sporozoites, Nitric oxide

## Abstract

Egress plays a vital role in the life cycle of apicomplexan parasites including *Eimeria tenella*, which has been attracting attention from various research groups. Many recent studies have focused on early egress induced by immune molecules to develop a new method of apicomplexan parasite elimination. In this study, we investigated whether nitric oxide (NO), an immune molecule produced by different types of cells in response to cytokine stimulation, could induce early egress of eimerian sporozoites *in vitro*. *Eimeria tenella* sporozoites were extracted and cultured in primary chicken kidney cells. The number of sporozoites egressed from infected cells was analyzed by flow cytometry after treatment with NO released by sodium nitroferricyanide (II) dihydrate. The results showed that exogenous NO stimulated the rapid egress of *E*. *tenella* sporozoites from primary chicken kidney cells before replication of the parasite. We also found that egress was dependent on intra-parasitic calcium ion (Ca^2+^) levels and no damage occurred to host cells after egress. The virulence of egressed sporozoites was significantly lower than that of fresh sporozoites. The results of this study contribute to a novel field examining the interactions between apicomplexan parasites and their host cells, as well as that of the clearance of intracellular pathogens by the host immune system.

## Abstract (fr)

La sortie des cellules joue un rôle vital dans le cycle de vie des parasites Apicomplexa, y compris *Eimeria tenella*, ce qui a attiré l’attention de plusieurs groupes de recherche. De nombreuses études récentes se sont concentrées sur la sortie précoce induite par des molécules immunitaires, pour développer une nouvelle méthode d’élimination des parasites Apicomplexa. Dans cette étude, nous avons examiné si l’oxyde nitrique (NO), une molécule immunitaire produite par différents types de cellules en réponse à la stimulation des cytokines, pouvait induire *in vitro* une sortie précoce des sporozoïtes des *Eimeria*. Les sporozoïtes d’*E. tenella* ont été extraits et cultivés dans des cellules primaires de rein de poulet. Le nombre de sporozoïtes sortant des cellules infectées a été analysé par cytométrie en flux après traitement avec du NO libéré par le nitroferricyanure de sodium (II) dihydraté. Les résultats ont montré que le NO exogène stimulait la sortie rapide des sporozoïtes d’*E. tenella* des cellules primaires de rein de poulet avant la réplication du parasite. Nous avons également constaté que la sortie dépendait des niveaux intra-parasitaires d’ions calcium (Ca^2+^) et qu’aucun dommage n’est survenu aux cellules hôtes après la sortie. La virulence des sporozoïtes sortis était significativement inférieure à celle des sporozoïtes frais. Les résultats de cette étude contribuent à un nouveau domaine d’étude des interactions entre les parasites Apicomplexa et leurs cellules hôtes, ainsi qu’à celui relatif à l’élimination des pathogènes intracellulaires par le système immunitaire de l’hôte.

## Introduction

Members of the Apicomplexan phylum are obligate intracellular pathogens and include *Toxoplasma gondii*, *Plasmodium* spp., and *Cryptosporidium* spp., which infect many vertebrate hosts including humans, causing severe disease [9, 25, 33]. Apicomplexan parasites undergo intricate processes in their asexual stages including invasion, replication, and egress [28]. Among these processes, egress is a vital step that enables parasites to invade another host cell for further development [14]. Egress usually results in lysis of the host cells, leading to deleterious consequences [5, 6]. As the process is crucial for both pathogens of this phylum and hosts, many studies have been conducted in this field [29, 30]. However, most of these reports are limited to *Plasmodium* spp. and *Toxoplasma gondii* [6]. *Eimeria* spp., is another important genus of apicomplexan parasites that causes severe negative impacts on the poultry industry worldwide, but little is known about the egress process of this genus [11].

*Eimeria* spp. can lead to severe intestinal disease in chickens, causing global poultry industry losses of approximately USD 3 billion per year [16, 31]. Understanding the relationship between *Eimeria* spp. and their host is of great interest for the control of coccidiosis. Our previous study revealed that calcium ions (Ca^2+^) and microneme 2 protein (EtMic 2) secretion were crucial for the egress of eimerian parasites [35, 36].

Many recent studies have focused on inducible egress stimulated by immune molecules to develop strategies for treating apicomplexan parasites [12, 27, 37]. For example, interferon-*γ*–induced cell death leads to early egress of *T*. *gondii* tachyzoites, which may disrupt the life cycle of host cells and promote elimination of this pathogen [26]. We recently reported that nitric oxide (NO) could stimulate the rapid egress of *T*. *gondii* tachyzoites from infected macrophages (immune cells) and human foreskin fibroblast cells (non-immune cells) [19, 34].

NO is produced by many cell types, such as dendritic, mast, natural killer, and phagocytic cells, in response to cytokine stimulation and plays important roles in immunologically mediated protection against many types of pathogens *in vitro* and in animal models, including protozoans [10, 17]. During infection by protozoans such as *Plasmodium* spp. and *Toxoplasma gondii*, increased NO production mediates host protection by either killing parasites directly or limiting parasite growth [8]. Previous studies have reported higher NO production during primary *E*. *tenella* infection, such that NO acts as an anticoccidial effector [1]. Therefore, we tried to detect whether NO could stimulate early egress of *E. tenella* sporozoites from host cells *in vitro* to supply more information for the study of *Eimeria* spp. egress and presented novel insights into the clearance of intracellular pathogens by hosts.

## Materials and methods

### Parasites, chickens and cell culture

The *E. tenella* M2e strain, a transgenic strain that stably expresses enhanced yellow fluorescent protein (EYFP) [21] used in this study, was maintained by propagating in coccidian-free Arber Acres broilers that were purchased from Beijing Arbor Acres Poultry Breeding Co., Ltd.; Beijing, China. All birds were housed in isolators to avoid contamination by other species of *Eimeria* spp. Isolation, purification, and sporulation of oocysts were conducted according to established protocols [22]. All animal experiments were conducted in accordance with animal ethics procedures approved by the China Agricultural University Laboratory Animal Welfare and Animal Experimental Ethical Inspection Committee (approval No. 20160921-2). Sporozoite purification was conducted as previously described [35]. Freshly purified sporozoites were suspended with phosphate-buffered saline (PBS) and the number of the parasites was counted using a blood cell counting plate. Primary chicken kidney cells (PCKs) were prepared as previously described [12] and seeded into 24-well culture plates supplemented with Dulbecco’s modified Eagle medium (DMEM) containing fetal bovine serum (10%, v/v), penicillin (200 U/mL), and streptomycin (100 μg/mL). The cell density was adjusted to 1 × 10^5^ cells/well.

### Measurement of NO concentration

The concentration of NO released by SNP was evaluated by measuring the NO_2_/NO_3_ ratio by Griess reaction using an NO assay kit (Applygen Tech Inc., Beijing, China), according to the manufacturer’s instructions.

### NO-induced egress assay

PCKs were cultured at 41 °C and 5% CO_2_ overnight followed by three washes with PBS to remove non-adherent cells. After 2 days, the PCKs were incubated with EtM2e sporozoites at a multiplicity of infection (MOI) of 2.0 (parasite/cell ratio of 2:1) at 37 °C for 12 h. Non-invasive sporozoites were removed by washing with PBS. The cells were then incubated with either various concentrations (10, 20, and 40 mM) of sodium nitroferricyanide (III) dihydrate (SNP; Sigma) or DMEM carrier alone for different treatment times (10, 20, and 30 min). After treatment, egressed parasites were suspended in 500 μL DMEM, and 10 μL suspensions were analyzed by flow cytometry (C6, Accuri Cytometers, Inc. Ann Arbor, MI, United States).

### Video microscopy

PCKs were treated with 40 mM SNP after 12 h of incubation with EtM2e sporozoites at an MOI of 2.0. Egress progression was recorded using a light microscope (Carl Zeiss, Olympus).

### Detection of effect on parasitic development stage

Before treatment with 40 mM SNP for 30 min, sporozoites were allowed to develop in PCKs for 24 or 36 h, which is the trophozoite stage of this parasite. After treatment, egressed parasites were suspended in 500 μL DMEM, and 10 μL suspensions were analyzed by flow cytometry.

### Determination of host cell viability

PCKs were washed after NO-induced egress, sporozoite invasion, SNP treatment and in untreated group, respectively, and cell viability was measured using propidium iodide (PI; eBioscience). Briefly, the PCKs in each group were washed with PBS and gently trypsinized after treatment, and single cells were stained with PI, according to the manufacturer’s instructions. The percentage of viable cells was analyzed by flow cytometry.

### Inhibition assay

For inhibition assays, the intra Ca^2+^ chelator BAPTA-AM (Sigma, St. Louis, MO, United States) and phosphoinositide specific phospholipase C inhibitor U-73122 (Sigma) were used to evaluate the effect of Ca^2+^ on sporozoite egress. In another experiment, cytochalasin D (CytoD; Merck, Darmstadt, Germany) was used to block parasitic motility. Infected PCKs were pre-treated with 10 μM BAPTA-AM, U-73122, or CytoD prepared in dimethyl sulfoxide (Sigma) for 30 min. Then, the cell cultures were incubated with 40 mM SNP for 30 min, and the number of egressed sporozoites was evaluated as described above.

### Virulence of egressed parasites

PCKs were incubated with sporozoites for 12 h followed by treatment with 40 mM SNP for 30 min. Cell culture medium containing free parasites after SNP treatment was collected and washed three times with PBS, and parasite viability was tested by using 4% trypan blue dye exclusion. The egressed parasites were counted using blood cell counting plates. To detect virulence, the re-invasion and reproductive abilities of egressed parasites after treatment by 40 mM NO for 30 min were tested. PCKs were incubated with egressed parasites at an MOI of 1:1 for 12 h, and the non-invasive parasites were suspended in 500 μL DMEM, and 10-μL suspensions were analyzed by flow cytometry. Freshly prepared sporozoites were used as a control. To evaluate the reproductive ability in the host, 2 × 10^4^ freshly prepared sporozoites and an equal quantity of egressed parasites were inoculated into 3-week-old AA broiler chickens through the cloacal route and all birds were housed in isolators [13]. The total output of oocysts per bird was measured using a McMaster egg counting chamber at 6–9 days after infection [15].

### Statistical analyses

SPSS 20.0 software (SPSS Inc.) was used to analyze differences between the treatment and control groups. All other statistical analyses were performed using Graphpad Prisma 5.01 software (Graphpad Software). Data are expressed as means ± standard error of the mean (SEM). Experimental groups were compared using one-way analysis of variance (ANOVA). Statistical significance was evaluated at levels of *p* < 0.05 (significant) and *p* < 0.01 (highly significant).

## Results

### Exogenous NO stimulated egress of *E. tenella* sporozoites

**Figure 1:**
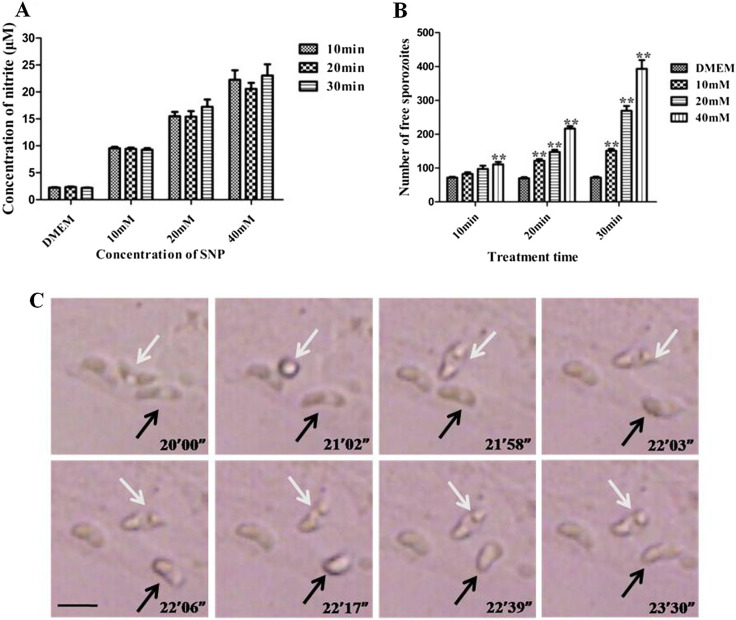
*Exogenous nitrous oxide (NO) stimulates egress of Eimeria tenella sporozoites*. (A) NO concentrations released by different doses of sodium nitroferricyanide (II) dihydrate (SNP) for a variety of durations. (B) Primary chicken kidney cells (PCKs) were infected with sporozoites and incubated with different concentrations of SNP for different times. The number of free sporozoites was analyzed by flow cytometry. Data are means ± standard error of the mean (SEM) of four replicates, representing three independent experiments. **p < 0.01, comparing SNP with DMEM at the respective time point. (C) Video was captured from 20 to 25 min after SNP treatment; eight representative frames were selected and shown. Arrows indicate the egress process in two sporozoites. Bar: 20 μm.

Before conducting egress assays, we verified whether the concentration of NO released from SNP could be kept stable when dissolved in DMEM within 30 min by Griess reaction (Fig. 1A). Then, we incubated sporozoite-infected cell cultures with different concentrations of SNP for different durations, as described above. The number of free sporozoites increased when the infected PCKs were incubated with SNP (Fig. 1B). We also observed the process of parasite egress by video recording (Fig. 1C).

### Effect of parasitic development stage on NO-stimulated egress

**Figure 2:**
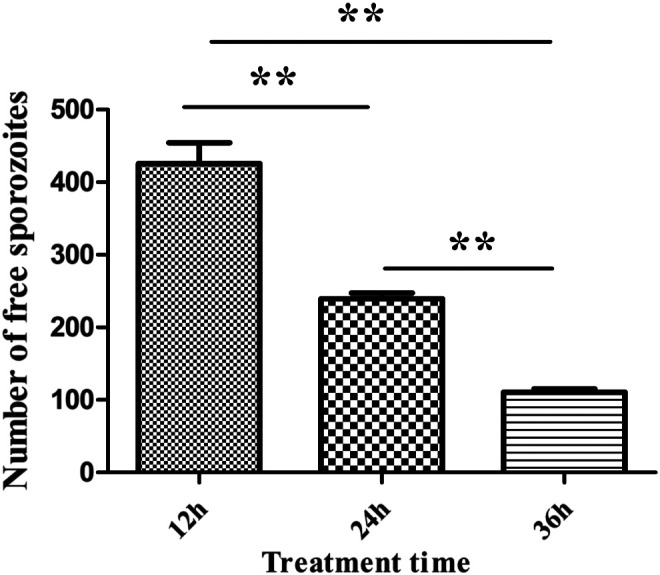
*Parasite development is essential for NO-induced egress*. PCKs were infected with sporozoites for 12, 24, or 36 h and then incubated with 40 mM SNP for 30 min. The number of free sporozoites was analyzed by flow cytometry. Data are means ± SEM of six replicates, representing three independent experiments. *p < 0.05; **p < 0.01.

We were interested in whether egress induced by NO could be influenced by the developmental stages of this parasite in PCKs. Before treating with 40 mM SNP for 30 min, we allowed the sporozoites to develop in host cells for 24 or 36 h, respectively, which is the trophozoite stage of this parasite. We surprisingly found that the number of egressed parasites was significantly decreased with extension of the development time of the parasite (Fig. 2), indicating that the developmental stage of the parasite is an important element of NO-induced egress.

### Egress of parasites dependent on parasitic Ca^2+^ and mobility

**Figure 3:**
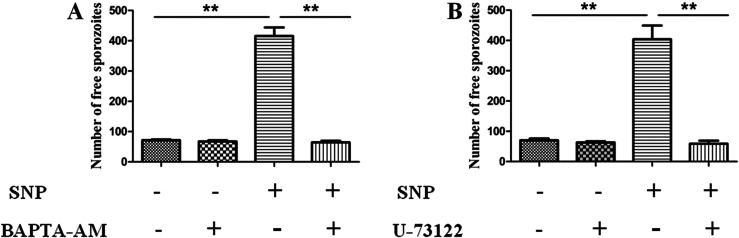
*NO-induced egress is required for intra-parasitic Ca* ^*2+*^. Sporozoite-infected PCKs were pre-treated with BAPTA-AM (A) or U-73122 (B) for 30 min and then incubated with 40 mM SNP for 30 min. Free parasites were analyzed by flow cytometry. Data are means ± SEM of six replicates, representing three independent experiments. **p < 0.01.

**Figure 4:**
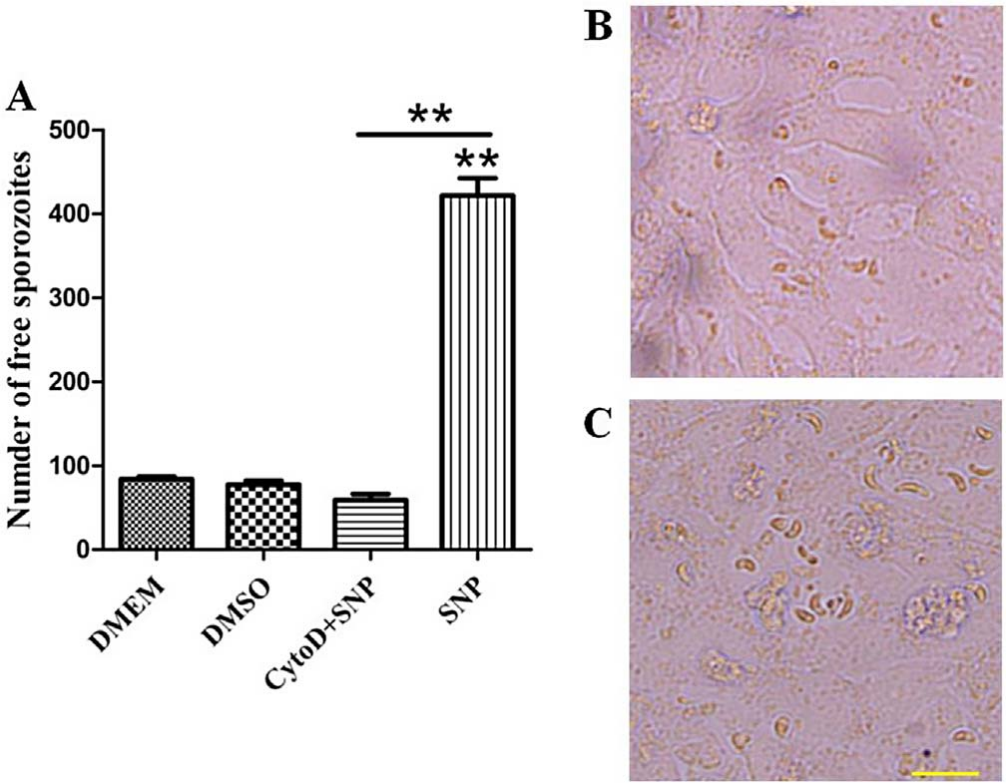
*NO-induced egress of sporozoites is dependent on parasitic mobility*. (A) Sporozoite-infected PCKs were pre-treated with 10 μM Cyto-D and then incubated with 40 mM SNP for 30 min. Free sporozoites were analyzed by flow cytometry. Data are means ± SEM of four replicates, representing three independent experiments. **p < 0.01. (B) and (C) Images captured after SNP treatment and pre-incubation with 10 μM Cyto-D. Bar: 20 μm.

A previous study showed that intra-parasitic Ca^2+^ played a vital role during the egress of *E*. *tenella* sporozoites [12, 34]. We performed experiments to determine the significance of Ca^2+^ in NO-induced egress of the parasite. As shown in Figures 3A and 3B, NO-induced egress was blocked by pre-treatment with both BAPTA-AM and U-73122, indicating the vital role of intra-parasitic Ca^2+^ and that the source of Ca^2+^ is the endoplasmic reticulum (ER). Moreover, pre-treatment with Cyto-D to block mobility of the parasite showed similar results (Figs. 4A and 4B), indicating that mobility of the parasite is another important factor for sporozoite egress.

### Parasite egress is not associated with host cell damage

**Figure 5:**
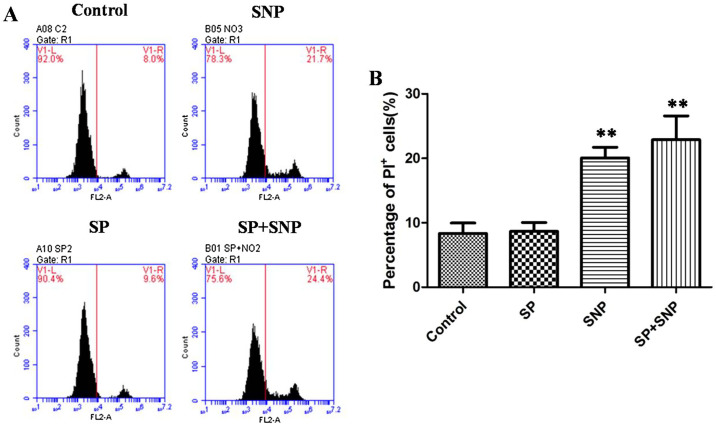
*NO-induced egress results in no host cell damage*. (A) and (B) Propidium iodide (PI) staining of PCKs in flow cytometry, representing percentages of PI^+^ cells after parasite invasion, SNP treatment, or SNP induced egress, no treatment as control. Representative data from one and three independent experiments are shown. Bars indicate means ± SEM (*n* = 5). **p < 0.01.

To investigate the possibility that NO-induced egress was toxic to the host cells, cell viability was measured by PI staining. The results showed that the percentage of PI^+^ cells increased after treatment with SNP, and there was no difference between non-infected and infected groups, indicating that incubation of SNP resulted in cell damage, but not parasite egress (Figs. 5A and 5B).

### Virulence of the parasite reduced after NO-induced egress

**Figure 6:**
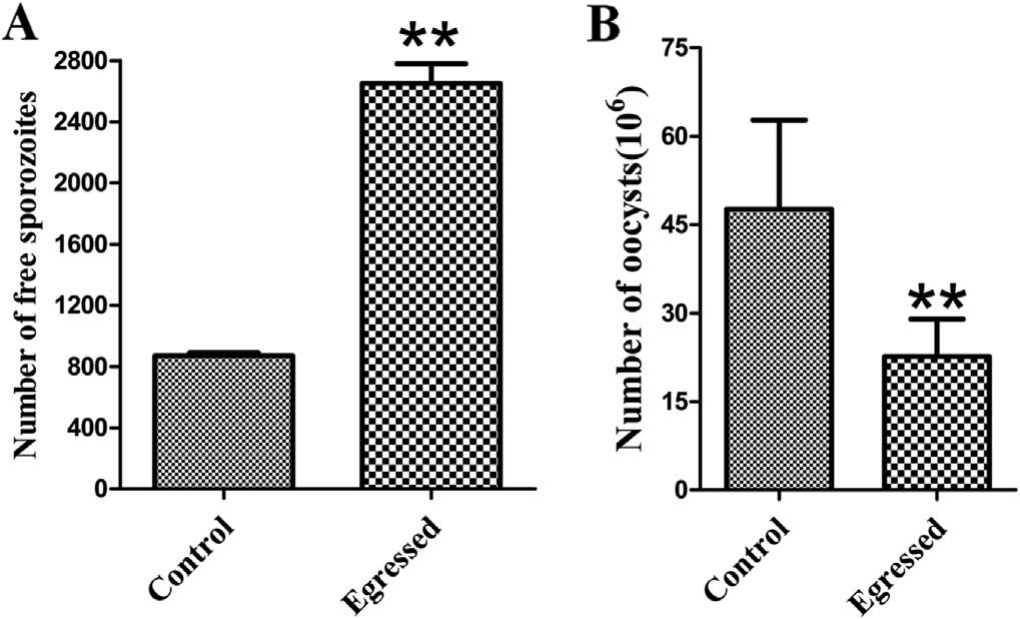
*Decreased virulence in egressed parasites*. Free parasites were collected after egress and counted using blood cell counting plates. (A) Egressed parasites were added to freshly prepared PCKs and incubated for 12 h; free sporozoites were suspended in 500 μL DMEM, and 10 μL suspensions were analyzed by flow cytometry after incubation. Data are means ± SEM of five replicates, representing three independent experiments. **p < 0.01. (B) Six birds were infected with egressed parasites (2 × 10^4^) via the cloacal route. Oocyst output per bird was quantified using a MacMaster chamber at 6–9 days after infection. Bars indicate means ± SEM. **p < 0.01.

Egressed parasites were collected and shown to remain viable by trypan blue exclusion (> 85% viability). Then we added the egressed parasites to PCKs or inoculated them into 3-week-old AA broiler chickens individually via the cloacal route. Both invasive efficacy (*p* < 0.01) (Fig. 6A) and reproductivity in chickens (*p* < 0.01) (Fig. 6B) of the parasites after NO-induced egress decreased dramatically compared to that of freshly prepared sporozoites.

## Discussion

In this study, we found that exogenous NO stimulated early egress of *E*. *tenella* sporozoites from infected PCKs in a Ca^2+^-dependent process. Parasite egress resulted in no damage of the host cells, and the re-invasive ability and virulence of the egressed parasites decreased significantly. Together, these results suggest a novel model for studying the mechanism of eimerian parasite egress and provide a new method, mediated by NO, to reduce intracellular pathogens in host cells.

Egress is an important process that enables eimerian parasites to exit host cells and invade neighboring cells to begin a new intracellular life cycle; thus, egress plays a crucial role in pathogenesis within the host. Our previous reports focused on preliminary mechanistic studies of eimerian parasite egress [35, 36]. In this study, NO was included. We found that NO could induce the egress of the parasites shortly after invasion completion, and long before the occurrence of mature merozoite egress, which disrupted the life cycle of this pathogen. In the present study, infected PCKs incurred no damage during egress, which differs from reports of host cell necrosis in similar studies of *T*. *gondii* tachyzoite egress [19, 20]. One plausible explanation for this difference is that the sporozoites did not replicate before the egress assay was conducted. Therefore, no more than two parasites inhabited the host cells, a value much lower than that of tachyzoites reported in previous studies [19, 32]. Another possible explanation is that, unlike *T*. *gondii* tachyzoites or eimerian merozoites, the parasite lacks a specific protein to destroy host cells for natural egress in the sporozoite stage. However, we found no direct evidence to support these hypotheses. Our results showed significant reduction in the virulence of egressed sporozoites, in contrast to *T*. *gondii* tachyzoites, which maintain normal virulence after egress [27, 37]. We assume that the parasite had been developing from sporozoites to schizonts when the egress assay was conducted, and that gene expression regulating stage-specific proteins in the parasite may have changed. In contrast to eimerian sporozoites, *T*. *gondii* tachyzoites undergo binary fission in host cells, thereby maintaining normal virulence. Therefore, we thought that if there were certain stimulators that could induce egress of *Eimeria spp*. after invasion then the egressed parasites could not maintain their normal virulence *in vivo*, like in *in vitro* experiments. This immune-mediated egress may disrupt the parasitic lifecycle and eventually promote clearance of the parasite by immune cells.

Recent studies have revealed the crucial role of Ca^2+^ in the egress of apicomplexan pathogens [7, 18]. The secondary messenger inositol 1,4,5-triphosphate (IP3) stimulates the release of Ca^2+^ from the ER into the cytoplasm through IP3 receptor binding [3]. IP3 has been shown to induce the release of Ca^2+^ from intracellular pools in both *T*. *gondii* [24] and *Plasmodium falciparum* [2]; however, little is known of the genomic presence of IP3 receptors in apicomplexan parasites [4, 23]. In the present study, intracellular Ca^2+^ and IP3 pathways were blocked using BAPTA-AM and U-73122 to test whether NO-induced egress of sporozoites is Ca^2+^-dependent and to determine the source of the Ca^2+^. Our results showed that egress was restrained by both BAPTA-AM and U-73122, indicating that intracellular Ca^2+^ plays a vital role in *E*. *tenella* sporozoite egress. Although we demonstrated that intracellular Ca^2+^ is vital to parasite egress, the pathway and the function of related proteins require further study using more advanced and precise methods.

In summary, the results of this study indicate that NO can stimulate early egress of *E*. *tenella* sporozoites from infected PCKs and provide preliminary information on the mechanism of this egress. In future studies, we will seek to identify additional immune molecules that induce early egress of *E*. *tenella* sporozoites and investigate whether NO can induce early egress of eimerian parasites from chicken intestinal epithelial cells *in vitro* and *in vivo*.

### Conflict of interest

The authors declare that they have no competing interests.
